# Getting up With Lateral Thinking

**DOI:** 10.1177/27536351241229952

**Published:** 2024-02-09

**Authors:** Anita Patel, Stephen Kirker

**Affiliations:** 1 School of Clinical Medicine, University of Cambridge, Cambridge, UK; 2 Cambridge University Hospitals NHS Foundation Trust, Cambridge, UK

**Keywords:** Falls, rehabilitation, case report, air mattress, rhabdomyolysis, pressure sores, Raizer chair, Mangar Elk, long lie

## Abstract

**Introduction:**

Falls in the community can have major impacts on patient lives. There can be long lasting physical and psychological consequences of a fall and subsequent long lie. The annual burden to ambulance services responding to falls at home is high. Affordable devices to help people get up from the floor, or reduce the risk of a long lie, would be useful and widely applicable.

**Case report:**

We present the case of 2 families who successfully used an air mattress and a bath lift to get the fallen person up off the floor following a fall, when they had previously called an ambulance. This has reduced their dependence on the ambulance service and has improved their confidence following falls.

**Discussion/conclusion:**

Affordable devices such as air mattresses can help people off the floor following a fall and prevent long lies as well as reduce the number of ambulance call outs.

## Introduction

Falls affect 30% of adults aged 65 and over every year and so constitute a major public health problem.^1^ The events following a fall can leave the person physically, emotionally and psychologically affected as well as placing considerable strain on the resources of ambulance services.^2^ Falls at home account for a significant proportion of ambulance call outs. Snooks et al^3^ found that 8% of all calls made to emergency services in London were due to older people who had fallen at home. 36% of these people did not require an admission to hospital. Here we will discuss the use of unconventional devices such as a blow-up air mattress and bath seat by 2 adult patients following a fall and the impact it has had on their lives.

## Case Reports

A 59-year-old gentleman with primary progressive multiple sclerosis had another fall in his own home in September 2021. He was unable to get himself up off the floor and the fall resulted in an injury to his cheek. His wife called the ambulance and was told it would take at least 4 hours for them to arrive as it was not an emergency.

As he previously had 12 to 15 falls, his wife had looked into options to help him off the floor after a fall but all of the available ones were too expensive. Her husband’s rehabilitation doctor had mentioned that a blow-up air mattress may work. His wife had subsequently purchased a queen size air mattress with a built-in electric pump online. After getting off the phone to the ambulance she transferred the mattress from the garage into the house, opened it out flat next to him, plugged it in and asked him to roll himself onto it. She then pressed the button to inflate the mattress and he was lifted 18 inches off the floor to a height where he could sit. Using his rollator frame, he had enough upper body strength to stand from the seated position and so they cancelled the ambulance call out. She later drove him to Accident and Emergency to have his injury looked at, however there were no concerns which required admission and he was sent home that same day.

He had another fall in June 2023 when he fell in his hallway. The air mattress was too large to inflate in the hallway, so he shuffled himself into the kitchen where his wife used the air mattress again to help him safely off the floor without any ambulance call out being made. She plans to order a single air mattress which would fit in the hallway and be better for falls in areas with limited space. She has also considered the use of a slide sheet to reduce the risk of carpet burn should her husband need to shuffle himself to the mattress in future.

The gentleman mentioned that the mattress has had a significant impact on his life and that an element of his confidence to keep walking post falls is knowing that he can use the air mattress to get up off the floor relatively quickly and does not have to stay on the floor for hours waiting for an ambulance to arrive. His wife also stated she feels more confident that she will be able to help him up following a fall now she has the air mattress. When asked to rate her confidence to lift her husband off the floor after a fall on a scale of 1 to 10 (10 being the highest) before versus after buying the mattress, she said it was 5 before compared to 8 now but would be even higher once the single mattress arrives. In the event his wife is not home during a fall, they have made provisions for his neighbour to bring the mattress to him.

Another method has been devised by a 66-year-old lady (with left hemiparesis following a spontaneous intracerebral haemorrhage) and her husband. She has had several falls since her diagnosis in 2020, most recently in September 2023. One day her husband thought to use the battery-operated bath lift to help her up off the floor. He placed it next to her flat on the floor, she shuffled herself onto it and pressed the switch which elevated her to chair height. From seated she could either stand or transfer onto her wheeled commode and go to an armchair or her bed. He has now used the bath lift to successfully help her up off the floor 6 times. Prior to using the bath lift, her husband would have to use unsafe techniques such as picking her up from under her arms which she described as painful or would have to call the ambulance. She has previously had to call the ambulance 3 times after a fall and was told that the wait could be up to 5 hours. She mentioned the chair is very sturdy and has back support which she finds comfortable. She feels her confidence to walk has increased by 100% since using the chair as she does not have the fear of lying on the floor for many hours whilst waiting for an ambulance.

Data provided by the East of England Ambulance service shows that between September to December 2022, 13 120 ambulance call outs were received for falls, of which 54% were classed as category 3 and had a mean response time of 215 minutes.^4^ (Mean response time is defined as the average time from clock start to scene arrival based on a mean average [total response time divided by number of incidents].) Calls which are marked as category 3 are urgent and should be responded to within 120 minutes according to the NHS England New Ambulance Standards.^5^ We believe patients in category 3 are the ones most likely to benefit from the devices mentioned.

## Discussion

The consequences of a fall include, but are not limited to, pressure sores (which can be made worse by unavoidable incontinence), carpet burns, dehydration, hypothermia, pneumonia and even death.^6^ The impact of a fall is made worse by a long lie which is defined as greater than 1 hour after a fall. A study of 125 adults over 65 years found that 50% of those who lay on the floor for more than 1 hour died within the following 6 months even if they were uninjured in the original fall.^7^ Given the potential catastrophic impacts of a long lie, it is essential individuals are lifted off the floor as quickly as possible after a fall. One of the biggest advantages of the air mattress over the other devices on the market is that even if the patient is unable to stand from the seated position, they are better off laying on an air mattress whilst waiting for an ambulance, rather than on the floor, as it lowers the risk of hypothermia, pressure sores, rhabdomyolysis and the long-term psychological loss of confidence due to fear of falling.

If the patient can stand from the seated position, the air mattress is also a good solution to help them back on their feet. Dining chairs are typically manufactured to a seat height of 17 to 19 inches from the floor. The air mattresses linked in the table below inflate to a maximum height of 18 to 20 inches bringing the individual to a comfortable seated position from which they may be able to stand.

Whilst there is equipment on the market specifically targeted at helping patients off the floor, they start from £500 and go up to £5500 depending on the make and model. At the upper end of this range is the Raizer chair which is a battery-operated mobile lifting chair. The main disadvantages are that it is an expensive piece of equipment and must be operated by a competent care professional. Another option is the Mangar Elk lifting cushion which has a similar mechanism to an electric air-mattress but as a medical device, it is much more expensive. One of the disadvantages of the Mangar Elk is that it has a small surface area and no back support so the patient cannot lie on it and would need to have sufficiently strong trunk and abdominal muscles to keep them in an upright position. Due to its small surface area, in contrast to the air mattress, it cannot be used as an interim option whilst waiting for an ambulance as it would be uncomfortable for someone to sit without any back support for prolonged periods.^8^ However, its smaller size does allow it to fit securely into small spaces such as hallways or bathrooms. A summary of the advantages and disadvantages of existing medical devices used for helping people up off the floor as well as the alternative devices used by the patients in our case reports is provided in Table 1.

**Table 1.** Medical devices used to help someone off the floor following a fall.

**Device name**	**Price (Incl. VAT)**	**Link to buy**	**Advantages**	**Disadvantages**
**Existing medical devices used**				
Raizer II lifting chair	£5479.99	https://www.medicalsupplies.co.uk/raizer-ii-emergency-patient-lifting-chair.html	• Remote controlled • Raises individual to almost standing in 30 s • Goes completely flat on ground so individual does not have to be able to sit	• Expensive • May be cost prohibitive to individuals at home
Raizer M manual lifting chair	£2345.99	https://www.medicalsupplies.co.uk/raizer-m-manual-emergency-patient-lifting-chair.html	• Manually operated and not reliant on batteries or electricity so can use it wherever needed • Goes completely flat on ground so individual does not have to be able to sit	• Expensive • May be cost prohibitive to individuals at home
Mangar elk lifting cushion	£1595.94	https://www.mobilitysmart.co.uk/mangar-elk-emergency-lifting-cushion.html	• Good for use in small and confined spaces	• Individual has to be able to get to seated position in order to slide onto cushion • For individuals who cannot go from sitting to standing the device cannot help them whilst waiting for an ambulance due to its small surface area and lack of back support
Portable hoist	£837.60	https://careoutlet.co.uk/invacare-birdie-evo-compact-hoist	• Device is on wheels so individual can be moved to another room	• Needs space to operate in and may require 2 fully trained caregivers • Not designed for long-term sitting so individual needs to be lowered to another chair or bed
Mangar bathing cushion	£599.00	https://www.boots.com/nrs-healthcare-mangar-inflating-raising-bathing-cushionand-compressor-grey-10320929	• Good for use in small and confined spaces • Cheaper alternative to the Mangar Elk lifting cushion • Multi-purpose can be used for baths	• Individual has to be able to get to seated position in order to slide onto cushion • For individuals who cannot go from sitting to standing the device cannot help them whilst waiting for an ambulance due to its small surface area and lack of back support
Alternative devices used in case reports				
Bellavita bath lift	£239.99	https://www.medicalsupplies.co.uk/470100312.html	• Good for use in small and confined spaces • Only weighs 9.3 kg, has a slim frame and is easy to move. The plastic build is sturdy and easy to clean • More affordable than the Mangar bathing cushion • Back support • Multi-purpose can be used for baths	• Individual has to be able to get to seated position in order to slide onto seat as backrest only reclines to 50° from the ground and does not go flat • For individuals who cannot go from sitting to standing it is not as comfortable to wait in the chair as it would be to lie on an air mattress, however they can transfer to another chair
Get fit airbed	£59.49	Get Fit Air Bed with Built In Electric Pump – Premium Single Size – Blow Up Bed with Free Pillow – Elevated Inflatable Air Mattress for Outdoor, Camping, Tents – Black/Grey : Amazon.co.uk: Home & Kitchen	• Inflates up to 20 inches in height • Can be used as interim option to lie on whilst waiting for ambulance to arrive • Individual does not have to be able to get to seated position as mattress rolls out flat on the floor • Affordable	• Requires space to operate
Airefina airbed	£79.99	Airefina Single Size Air Bed with Built-in Electric Pump, Inflatable bed in 3 Mins Self-Inflation/Deflation, Air Mattress Flocked Surface Blow Up Bed for Home Camping 190 × 99 × 46 cm, 295 kg MAX : Amazon.co.uk: Home & Kitchen	• Inflates up to 18 inches in height • Can be used as interim option to lie on whilst waiting for ambulance to arrive • Individual does not have to be able to get to seated position as mattress rolls out flat on the floor • Affordable	• Requires space to operate
Bestway airbed	£69.99	Bestway King Queen Double Single Size Air Bed | Airbed with Built-in Electric Pump, Fast Inflation, Wave Beam Support Mattress with Storage Bag, Grey : Amazon.co.uk: Home & Kitchen	• Inflates up to 18 inches in height • Can be used as interim option to lie on whilst waiting for ambulance to arrive • Individual does not have to be able to get to seated position as mattress rolls out flat on the floor • Affordable	• Requires space to operate
Olarhike airbed	£67.99	OlarHike Single Airbed, Inflatable Mattress with Built-in Electic Pump, Self-inflating Folding Guest Airbed, Inflatable Air Mattresses For Camping or Home Use with Storage, 198 × 102 × 46 cm (Black) : Amazon.co.uk: Sports & Outdoors	• Inflates up to 18 inches in height • Can be used as interim option to lie on whilst waiting for ambulance to arrive • Individual does not have to be able to get to seated position as mattress rolls out flat on the floor • Affordable	• Requires space to operate
Green haven airbed	£49.99	Green Haven Premium Large Single Size Air Bed with Rapid Built in Electric Pump & Storage Bag – Raised Flocked Airbed with a Soft Top | Fast Self-inflating Camping Bed with Upgraded Internal Beams : Amazon.co.uk: Home & Kitchen	• Inflates up to 18 inches in height • Can be used as interim option to lie on whilst waiting for ambulance to arrive • Individual does not have to be able to get to seated position as mattress rolls out flat on the floor • Affordable	• Requires space to operate

The retail price, advantages and disadvantages of each device are included.

Whilst this report focuses on the aftermath of a fall rather than prevention, it goes without saying that unexpected and sudden falls should not be prematurely labelled as mechanical falls and the use of these devices are not a replacement for proper follow-up or medical review where required. In both cases, family members and/or neighbours applied a commonsense approach to dealing with a fall. However, we appreciate that many people will rely on care professionals to support them in the community rather than family members. In these instances, we anticipate stringent guidelines, training and competency needs, health and safety concerns and fear of litigation may delay the adoption of these techniques by professional carers.

A key principle of rehabilitation medicine is to encourage individuals with life-long chronic and debilitating conditions to be self-reliant. The process of using a blow-up air mattress or bath lift to elevate a patient off the ground is technically simple, cost effective and can be used in the patients’ own home by family members, care professionals and neighbours.

In summary, current devices on the market to help vulnerable adults off the floor following a fall are expensive and therefore inaccessible to many. Unconventional techniques to aid people off the floor, such as the use of blow-up air mattresses and bath lifts, may reduce the incidence of long lies, dampen the psychological impacts of a fall and may reduce the number of ambulance call outs.
